# Clinical Research in the Hospital During the Pandemic: What’s Worked and Not Worked?

**DOI:** 10.1093/geroni/igab046.1204

**Published:** 2021-12-17

**Authors:** Marie Boltz, Ashley Kuzmik, Irene Best, Jacqueline Mogle

**Affiliations:** 1 Pennsylvania State University, University Park, Pennsylvania, United States; 2 Penn State University, University Park, Pennsylvania, United States

## Abstract

Under normal conditions, the hospital setting presents multiple challenges to research with persons with dementia and their care partners. This presentation describes the additional barriers posed by the COVID-19 pandemic, as well as the strategies to meet those challenges, in a cluster randomized controlled trial that examines the efficacy of a nurse-family partnership to promote functional recovery of persons with dementia. In response to research restrictions, the research team altered their plan for recruitment, implementation of the intervention, data collection, and analytic approach. This presentation describes these alterations and discusses the plan to meet the aims of the project while meeting the requirements of the Institutional Review Board, accountability to the funder, and university regulations. Modifications in staffing patterns, staff training, and procedures will also be discussed, as well as the study timeline. Finally, strategies to maintain a positive attitude and productivity within the team will be discussed.

